# Superposition of Automatic and Voluntary Aspects of Grip Force Control in Humans during Object Manipulation

**DOI:** 10.1371/journal.pone.0079341

**Published:** 2013-11-11

**Authors:** Frederic Danion

**Affiliations:** Institut de Neurosciences de la Timone, CNRS & Aix-Marseille University, Marseille, France; Ben-Gurion University of the Negev, Israel

## Abstract

When moving grasped objects, people automatically modulate grip force (GF) with movement-dependent load force (LF) in order to prevent object slip. However, GF can also be modulated voluntarily as when squeezing an object. Here we investigated possible interactions between automatic and voluntary GF control. Participants were asked to generate horizontal cyclic movements (between 0.6 and 2.0 Hz) of a hand-held object that was restrained by an elastic band such that the load force (LF) reached a peak once per movement cycle, and to simultaneously squeeze the object at each movement reversal (i.e., twice per cycle). Participants also performed two control tasks in which they either only moved (between 0.6 and 2.0 Hz) or squeezed (between 1.2 and 4.0 Hz) the object. The extent to which GF modulation in the simultaneous task could be predicted from the two control tasks was assessed using power spectral analyses. At all frequencies, the GF power spectra from the simultaneous task exhibited two prominent components that occurred at the cycle frequency (ƒ) and at twice this frequency (2ƒ), whereas the spectra from the movement and squeeze control task exhibited only single peaks at ƒ and 2ƒ, respectively. At lower frequencies, the magnitudes of both frequency components in the simultaneous task were similar to the magnitudes of the corresponding components in the control tasks. However, as frequency increased, the magnitudes of both components in the simultaneous task were greater than the magnitudes of the corresponding control task components. Moreover, the phase relationship between the ƒ components of GF and LF began to drift from the value observed in the movement control task. Overall these results suggest that, at lower movement frequencies, voluntary and automatic GF control processes operate at different hierarchical levels. Several mechanisms are discussed to account for interaction effects observed at higher movement frequencies.

## Introduction

The interplay between automatic and voluntary mechanisms is crucial and obvious in many motor behaviours. It has been investigated in the context of walking [1]–[4], swallowing [5], [6], breathing [7]–[9], reaching [10]–[12], and postural control (for reviews see [13], [14]). In this paper we examine the interplay between automatic and voluntary grip force control mechanisms in object manipulation, using a task in which automatic and voluntary modulations in grip force are generated simultaneously.

On the one hand, it is obvious that grip force can be under voluntary control, as when, for example, we want to squeeze an object to break or deform it [15]. Explicit control of grip force has been investigated in tasks requiring participants to modulate grip force so as to track or reach a force level target [16]–[19]. On the other hand, there are also cases in which grip force is less explicitly controlled. This is typically the case when we lift and/or transport an object and modulate grip force in synchrony with movement-dependent load forces in order to stabilize the object [20], [21]. Because participants are rarely explicitly aware of this coupling between grip force and load force [22], it is often viewed as an automatic process. Not only is this coupling robust to changes in experimental conditions [23]–[28], but it is also hard to suppress [22]. Indeed when participants move an object and are asked to maintain a constant grip force [22], or to employ unnecessary high grip force [29], the grip-load force coupling persists.

**Figure 1:**
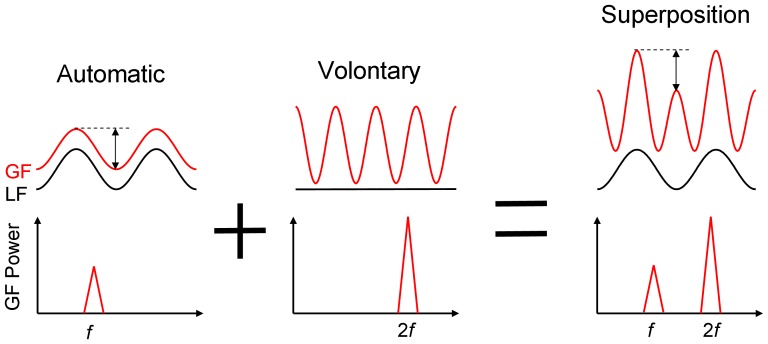
Hypothetical grip force (GF) and load force (LF) signals when oscillating or squeezing a hand-held object, as well as when performing both tasks simultaneously (algebraic summation). The top row presents the temporal dynamics of the force signals in each condition, and the bottom row presents the associated power spectrum of GF.

The goal of the current study was to investigate the extent to which automatic and voluntary grip force control can operate independently [grip force reflexes triggered by unexpected perturbations also fall under the category of automatic processes, but those will not be addressed here]. Participants were required to oscillate a hand-held object that was restrained by an elastic band while simultaneously squeezing the object at each movement reversal. Participants also performed two control tasks in which they only moved the object or only squeezed the object. If automatic and voluntary modulations of grip force are produced independently, we expect the time-varying grip force seen in the combined task to equal the sum of the time-varying grip forces seen in the two control tasks. The extent to which such superposition occurs can be evaluated in three different ways. The left side of Figure 1 shows hypothetical grip force and load force signals that correspond to either oscillating an object against an elastic load at frequency *f*, or maintaining the object steady while squeezing it rhythmically at 2*f*. The algebraic summation of both tasks is shown on the right side of the figure. If automatic and voluntary processes can be smoothly superimposed, a first prediction is that the power spectrum of the grip force signal in the combined task should exhibit two peaks whose magnitude is similar to those observed when performing each task in isolation. A second prediction is that smooth superposition should not alter the phase relationship between the automatic grip force component and the load force (i.e. grip-load force coupling), so that it remains rather close to zero. Finally, a third prediction is that smooth superposition should lead to an asymmetry between successive peaks, such that higher grip force peaks will be reached when voluntary pulses are initiated in conjunction with a high tension of the elastics (the magnitude of the asymmetry should match the amplitude of the automatic component). Because the risk of interference when superimposing two motor actions over the same limb has been shown to depend on the temporal constraints of the task [30], [31], we investigated a range of movement frequencies. In one additional experiment, carried out to follow up on questions arising from the first, we (1) replicated the first experiment while minimizing the contribution of automatic grip force control (by disconnecting the elastic band from the object and attaching it to the wrist), and (2) examined a bimanual version of the combined task in which subjects had to oscillate the object with one hand while generating voluntary pulses with the other hand.

## Materials and Methods

### Participants

Forty healthy participants (26 males and 14 females) took part in this study after giving written informed consent. The study was approved by the local ethics committee from the Aix-Marseille University. The experiment was conducted in accordance with the Declaration of Helsinki. Participants were split in two groups of 20 (Group 1 mean age  =  29 yr, Group 2 mean age  =  24 yr), with each group performing a different experiment. All participants were right-handed according to their preferential use of the right hand during writing and eating.

### Apparatus

**Figure 2:**
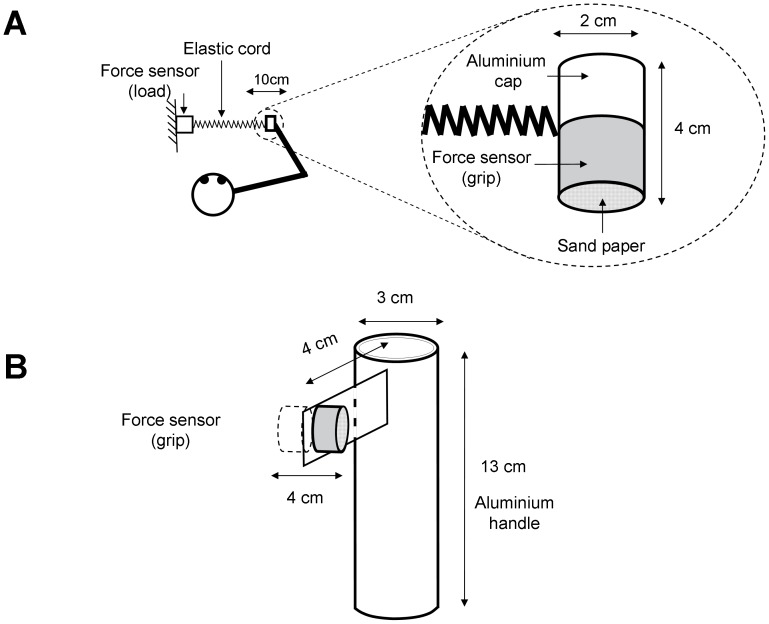
Experimental setup. A. Schematic drawing of the apparatus and of a participant holding the grasping device (top view). B. Schematic drawing of the handle grasped in the WRIST conditions in Experiment 2.

**Figure 3:**
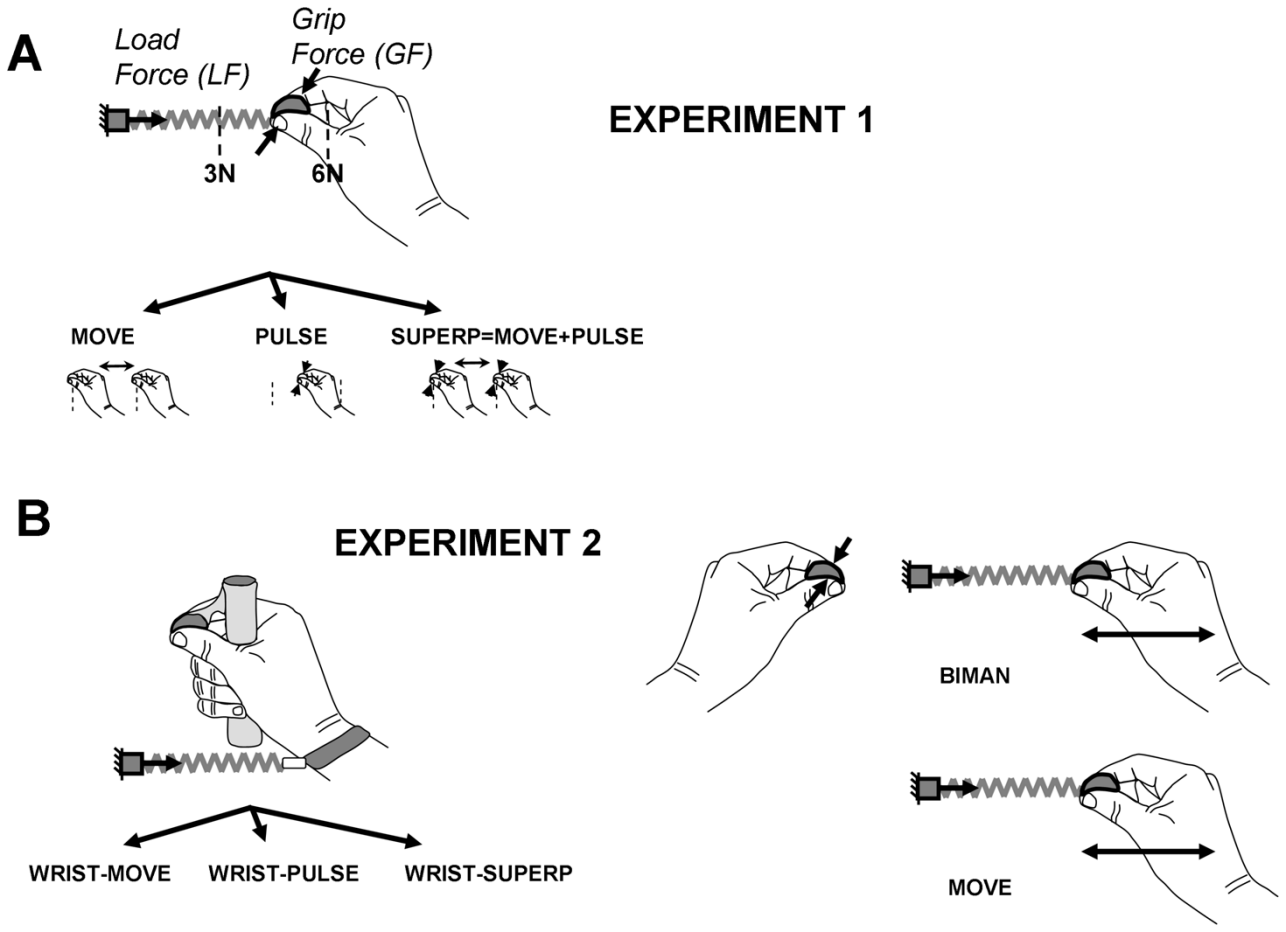
Experimental tasks. A. Schematic drawing illustrating the three experimental tasks used in Experiment 1. During MOVE, the participant had to oscillate the object between two targets. During PULSE, the participant had to squeeze rhythmically the object while keeping the object steady between the targets. During SUPERP, the participants had to simultaneously oscillate and squeeze the object. B. Same as A for the five experimental tasks used in Experiment 2. The first three tasks (left panel) were the replication of the tasks tested in Experiment 1 with the elastic band disconnected from the object and attached to the wrist. The two remaining tasks (right panel) consisted in the replication of the MOVE task, and a bimanual version of the SUPERP task. See Methods for further details.

The lightweight hand-held object (45 g) shown in Figure 2A contained a single force sensor (ELPM-T1M-50N, Entran, Fairfield, NJ) that measured the fingertip force applied perpendicularly to the sensor’s surface. The flat grip surfaces were covered by sandpaper. The object was attached to an elastic cord (stiffness  =  30 N/m) such that the load force (and minimum grip force to prevent slip of the object) increased linearly, from approximately 3 to 6 N, as a function of movement amplitude from the start position. The correlation between hand position and elastic tension in the workspace was above 0.99 (for a similar setup see [24], [32]. The other end of the elastic cord was attached to another force sensor (ELPM-T1M-25N, Entran, Fairfield, NJ), which measured both the load force and hence the position of the object. In some trials the instrumented object was attached to the top of a lightweight handle (20 g; see Figure 2B). During these trials participants securely held the handle using a power grip involving the middle, ring, and little fingers while grasping the instrumented object between the tips of the index finger and thumb (see left side of Figure 3B). Finally during some bimanual trials, a third force sensor (ELPM-T1M-50N, Entran, Fairfield, NJ) was used to measure grip force exerted by the non-moving hand (see right side of Figure 3B). The output of all the sensors was sent to a multi-channel signal conditioner (MSC12, Entran, Fairfield, NJ). The accuracy of the force sensors was 0.02 N. Using customized software (Docometre) and a real-time acquisition system (ADwin-Pro, Jäger, Germany), signals from each sensor were collected at the sampling frequency of 1000 Hz.

### Procedure

During testing, the participant sat on a chair whose position was adjusted at the beginning of the experiment to allow comfortable arm and trunk posture throughout the experimental sessions (see a top view in Figure 2A).

**Experiment 1.** Using the right hand, the participants grasped the instrumented object between the thumb and the index. Three different tasks were investigated (see Figure 3A). In the MOVE task, participants had to oscillate the instrumented object between two targets that were 10 cm apart and located at positions corresponding to load forces of 3 and 6 N. The movement had to be performed along a horizontal axis parallel to the participant’s frontal plane. Participants were instructed to synchronize movement reversals in the vicinity of the targets with the beeps of the metronome (i.e. movement frequency being half of the metronome frequency). In the PULSE task, participants had to produce rhythmical grip force pulses while maintaining the object stationary midway between the two targets and hence with a load force of 4.5 N. Participants were instructed to produce one grip force pulse at each beep of the metronome. No explicit instruction was given with regard to the amplitude of this pulse, but participants were encouraged to generate pulse with an amplitude they felt comfortable maintaining over several trials. In the SUPERP task, participants had to simultaneously perform the MOVE and the PULSE tasks. Specifically, they were instructed to oscillate the object while generating a grip force pulse in the vicinity of each target (i.e. at each metronome beep).

For each task, participants performed one block of 8 trials with metronome frequencies ranging from 1.2 Hz to 4 Hz in steps of 0.4 Hz. In the MOVE and SUPERP tasks, this resulted in target movement frequencies (*f*) of 0.6, 0.8, 1.0, 1.2, 1.4, 1.6, 1.8, and 2.0 Hz. In the PULSE and SUPERP tasks, this resulted in target pulse frequencies (2*f*) of 1.2, 1.6, 2.0, 2.4, 2.8, 3.2, 3.6, and 4.0 Hz. The order of the metronome frequencies was randomized for each task and each participant. The order of blocks was pseudo-randomized and counterbalanced across participants. Each trial lasted 12s. Prior to each block of 8 trials, each participant performed a few practice trials at low and intermediate frequencies. When participants failed to maintain the coordination imposed by the metronome, the trial was repeated. Participants were not provided with information about suitable grip forces or strategies on how to perform the tasks. Whenever necessary, rest periods were provided to prevent possible effects of fatigue. Overall the duration of Experiment 1 was about 50 minutes per participant.

Once the main experimental conditions described above had been completed, participants performed three post-experimental trials in order to evaluate the minimal ratio of GF to LF needed to prevent the object from slipping. During these trials, the object had to be maintained between the two targets (LF  =  4.5 N) and participants were asked to gradually release their grip until the object slipped. Initiation of slipping was taken as the time at which the rate of change of load force decreased below -2 N/s [33], [34]. The ratio of grip force to load force at that specific time represents the minimum ratio and was 0.52±0.10, averaged across participants.

**Experiment 2.** Five different tasks were investigated (see Figure 3B). The goal of the first three tasks was to investigate whether the superposition between automatic and voluntary GF control was directly responsible for alterations observed during Experiment 1. To achieve this goal the three tasks investigated in Experiment 1 were replicated while removing the contribution of automatic grip-load force coupling. Practically, the elastic band was disconnected from the object and attached directly to the wrist, and the instrumented object was mounted on the handle (see left side of Figure 3B). This enabled the participant to accelerate the object by applying forces to the handle, and without applying appreciable load forces to the object. In the WRIST-MOVE task, participants had to oscillate the instrumented object between the two targets in synchrony with the beeps of the metronome (as in MOVE). Participants were encouraged to maintain a low grip force on the force sensor and were successful in complying with this instruction (mean GF  =  1.1±0.5 N, SD across participants). In the WRIST-PULSE task, participants had to produce rhythmical grip force pulses while maintaining the object stationary midway between the two targets (as in the PULSE task). In the WRIST-SUPERP task, participants had to simultaneously squeeze and oscillate the object (as in the SUPERP task). Two additional tasks in which the elastic band was connected to the object were also tested (as in Experiment 1; see right side in Figure 3B). During the BIMANUAL task, participants had to simultaneously oscillate the object with the right hand, while generating grip force pulses with the left hand. The goal of this task was to investigate possible alterations in grip-load force coupling induced by voluntary GF control in the other hand. To allow direct comparison between Experiments 1 and 2, as well as to offer a baseline for interpreting grip-load force coupling results in the BIMANUAL task, the MOVE task was repeated in Experiment 2.

For each task, participants performed one block of 4 trials with metronome frequency ranging from 1.2 Hz to 3.6 Hz in steps of 0.8 Hz. During the WRIST-MOVE, WRIST-SUPERP, BIMANUAL and MOVE tasks, this resulted in target movement frequencies of 0.6, 1.0, 1.4, and 1.8 Hz. During WRIST-PULSE, WRIST-SUPERP and BIMANUAL (left hand) tasks, this resulted in target pulse frequencies of 1.2, 2.0, 2.8, and 3.6 Hz. As in Experiment 1, the order of the frequencies was randomized for each block and participant. The order of blocks was pseudo-randomized and counterbalanced across participants. Each trial lasted 12s. Initiation of slipping was also evaluated with three post-experimental trials. Over the group of participants, the minimum ratio of GF to LF was 0.60±0.11. Overall the duration of Experiment 2 was about 60 minutes per participant.

### Data analysis

The force signals were first smoothed using a fourth-order dual-pass Butterworth digital filter with a low-pass cut-off frequency of 20 Hz. Because we were interested in steady-state cyclic behaviour, the first 2s of each trial were discarded from the analysis (for rather similar procedures see [29], [35]). Fast Fourier Transforms (FFTs) performed over the last 10 s of each trial were used to assess the frequency content of the GF signal. Focus was on the amplitude of the two main FFT components: at movement frequency (GF*_f_*) and at twice the movement frequency (GF*_2f_*). To monitor the GF-LF coupling, the phase relation between GF and LF signals at movement frequency was also assessed by FFT (a positive phase lag indicating that GF precedes LF). Lastly, to investigate the asymmetry of GF peaks in the combined tasks, we compared the magnitudes of the GF peaks closest to LF maxima (GF_max1_) and the GF peaks closest to LF minima (GF_max2_). Repeated measures ANOVAs were used to assess the effects of task (*task)* and movement frequency (*f*). Post-hoc tests (Newman Keuls) were used where appropriate. All tests were performed with p < 0.05 as the significance criterion.

## Results

### Experiment 1

**Task performance.** Overall, participants performed fairly accurately the prescribed frequencies for the oscillatory movement and/or the force pulses. The mean absolute error between the prescribed movement frequency (ƒ) and the peak LF frequency was below 1% (error computed across all participants and all trials involving movement). Likewise, the mean absolute error between the peak GF frequency close to 2ƒ was below 1% (error computed across all participants and all trials involving pulses). The average LF amplitude (maximum minus minimum), based on participant means, was respectively 3.36 and 3.41 N in the SUPERP and MOVE conditions, but below 0.1 N in the PULSE condition meaning that participants had the hand fixed. The average LF was respectively 4.46, 4.52, 4.51 N in the SUPERP, MOVE and PULSE conditions. Overall those values suggest that participants complied well with the instructions.

**Figure 4:**
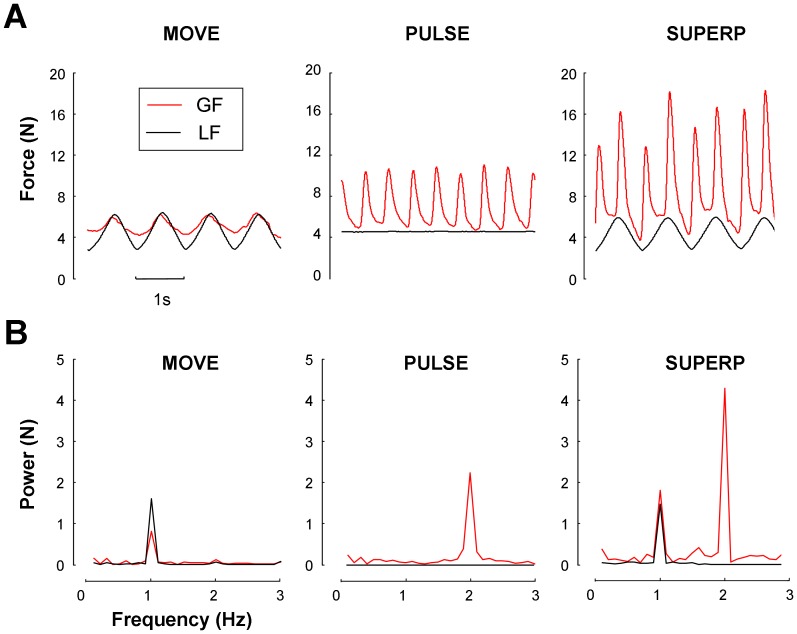
Typical trials in each experimental condition of Experiment 1. A. The top row presents the temporal dynamics of grip force and load force signals. B. The bottom row presents the associated power spectrum of each signal in each condition. All trials were performed by the same participant. Movement and squeezing frequency was set respectively at 1

**Power spectral analyses of GF.** Typical trials by the same participant in each experimental condition are displayed in Figure 4. As expected, when the object was oscillated at 1 Hz (MOVE), the power spectrum of GF and LF showed one major peak at that particular frequency. Similarly, when the object was immobile and squeezed rhythmically at 2 Hz (PULSE), the power spectrum showed a peak at 2 Hz for GF (with no visible peak for LF). When these two tasks were performed simultaneously (SUPERP), the power spectrum of GF carried two peaks, one at the movement frequency (1 Hz) and the other at the frequency of voluntary squeezing (2 Hz). Visual inspection of those spectra reveals that the magnitudes of the two GF peaks in the SUPERP task were larger than the corresponding single peaks in the MOVE and PULSE tasks.

**Figure 5:**
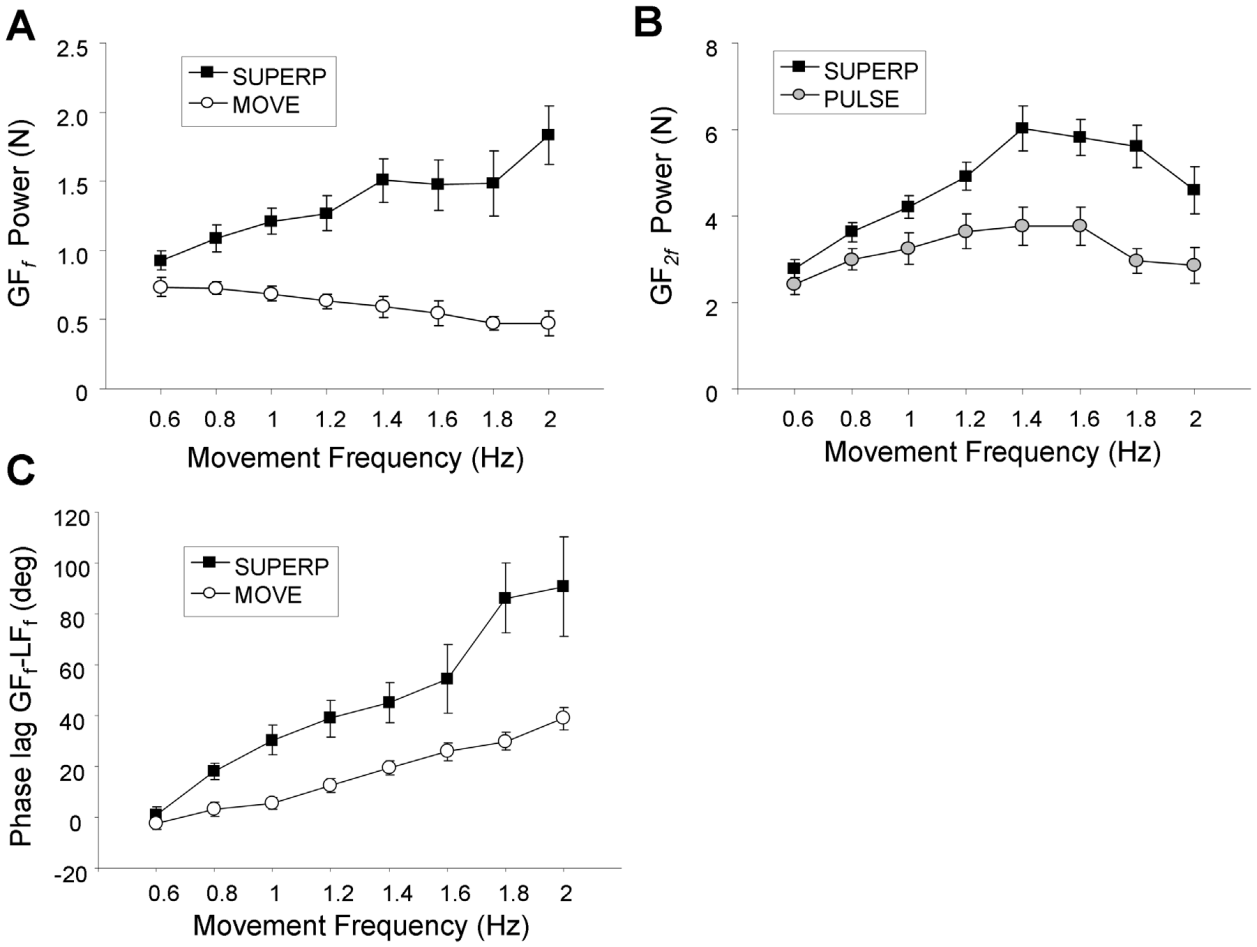
Spectral analysis of grip force signals in Experiment 1. A. Power of grip force at movement frequency (GF*_f_*) during the SUPERP and MOVE tasks. B. Power of grip force at twice the movement frequency (GF*_2f_*) during the SUPERP and PULSE tasks. C. Phase lag between grip force and load force at movement frequency during the SUPERP and MOVE tasks. A positive phase lag indicates that GF precedes LF. For all panels, data are averaged across participants, with error bars representing the standard error of the mean.

Figure 5A compares, as a function of movement frequency (*f*), the mean group GF power at movement frequency (GF*_f_*) in the SUPERP and MOVE tasks. Although the magnitude of GF*_f_* decreased as a function of *f* in MOVE, it increased substantially in SUPERP and approximately doubled between *f* = 0.6 Hz and *f* = 2 Hz. This resulted in a significant *task* by *f* interaction (F(7,133) = 11.86; p<0.001). Post-hoc analysis of this interaction revealed that, starting from *f* = 0.8 Hz, GF*_f_* was greater in SUPERP compared to MOVE (p<0.01). Main effects of *f* and *task* were both significant (p<0.01).

Figure 5B compares, as a function of movement frequency (*f*), the mean group GF power at twice the movement frequency (GF*_2f_*) in the SUPERP and PULSE tasks. As in the previous case, the two curves diverged substantially as *f* increased. Although both curves increased as a function of *f*, power increased more rapidly in SUPERP than in PULSE. This discrepancy was supported by a *task* by *f* interaction (F(7,133) = 5.73; p<0.001). Post-hoc analysis revealed that, starting from *f* = 1.0 Hz and above, GF_2*f*_ was greater in SUPERP compared to PULSE (p<0.05). Main effects of *f* and *task* were both significant (p<0.001). Overall this comparison between SUPERP and the two control tasks indicates that, beyond certain movement frequencies, both types of GF modulations were amplified when participants performed both tasks simultaneously.

**Figure 6:**
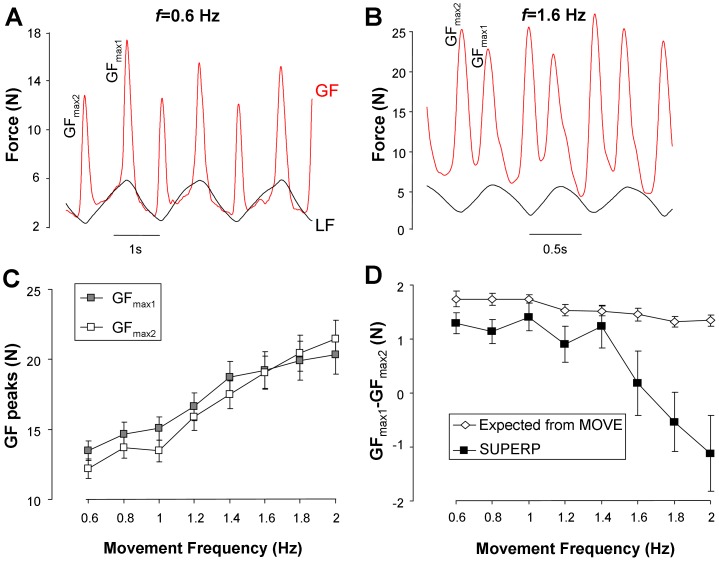
Asymmetry in grip force peaks during SUPERP. A. Grip force and load force signals in one representative trial during SUPERP at low movement frequency. B. Same as A but for a trial performed at high movement frequency (same participant). C. Analysis of grip force peaks asymmetry during SUPERP. This panel compares the magnitude of grip force peaks in the vicinity of load force maxima (GF*_max1_*) with those obtained in the vicinity of load force minima (GF*_max2_*). D. Comparison between the observed and expected asymmetry in grip force peaks (see text for more details). Data are averaged across participants, with error bars representing the standard error of the mean.

**Phase relationship between GF and LF during MOVE and SUPERP.** Two typical SUPERP trials performed by the same subject at *f* = 0.6 and *f* = 1.6 Hz are presented respectively in Figure 6A and B. In both cases one can distinguish a GF component (GF*_f_*) that mimics changes in LF. However, although the synchrony between these two components is obvious at low movement frequency, it is less obvious at higher movement frequency. Indeed in the latter case GF*_f_* is clearly leading on LF. To evaluate the robustness of this observation, the phase lag between GF*_f_* and LF*_f_* was computed in all trials. Figure 5D presents the evolution of this phase lag as a function of movement frequency in both the MOVE and SUPERP tasks. In agreement with the hypothesis of a smooth superposition between automatic and voluntary processes, at low movement frequency, the phase lag was low and similar in MOVE and SUPERP. However as movement frequency increased, the phase lag also increased, albeit more rapidly in the SUPERP task. These observations were confirmed by a two-way ANOVA showing main effects of *task* (F(1,19) = 20.35; p<0.001) and *f* (F(7,133) = 19.03; p<0.001), as well as *task* by *f* interaction (F(7,133) = 4.23; p<0.001). Post-hoc analysis of the interaction revealed that, starting from *f* = 1.2 Hz, the phase lag was greater in SUPERP compared to MOVE (p<0.05). Overall this analysis showed that, as movement frequency increased, the timing between GF and LF signals became altered in the superposition task.

**Asymmetry of GF peaks during SUPERP.** In agreement with the scheme of a smooth superposition, visual inspection of the SUPERP trial in Figure 6A shows that, at low movement frequency, GF peaks closest to LF max (when LF≈6 N) dominates over GF peaks closest to LF min (when LF≈3 N). However visual inspection of the SUPERP trial in Figure 6B suggests the opposite trend at higher movement frequency, with GF peaks at LF min dominating over GF peaks at LF max. To circumvent more rigorously this switch in behaviour, Figure 6C presents the mean group value of GF peaks at LF_max_ (GF_max1_) and LF_min_ (GF_max2_) as a function of movement frequency. Two-way ANOVA with *f* and *peak* (GF_max1_ vs GF_max2_) revealed a significant interaction (F(7,133) = 8.67, p<0.001). Post-hoc analysis of this interaction showed that, up to 1.4 Hz, GF_max1_ was significantly greater than GF_max2_ (p<0.001), whereas at 2.0 Hz, it was the other way around (p<0.01). The main effect of *f* was significant (F(7,133) = 26.95, p<0.001) as well as the main effect of *peak* (F(1,19) = 6.36, p<0.05).

To further determine whether the magnitude of this asymmetry in GF peaks was consistent with our hypothesis of linear summation, Figure 6D compares the difference between GF_max1_ and GF_max2_, with the peak-to-peak amplitude of GF oscillation during the MOVE task. Assuming that LF oscillations are the main source of asymmetry between GF peaks during SUPERP, it was predicted that GF_max1_ - GF_max2_ ≈+1.5 N. Although this scheme is well supported by the experimental data at low and intermediate movement frequencies, this is not the case at the highest movement frequencies. A two-way ANOVA with *task* (expected from MOVE vs SUPERP) and *f* showed an interaction (F(7,133) = 5.86, p<0.001). The post-hoc analysis showed that, starting from *f* = 1.6 Hz and above, there was a significant difference between the observed and expected asymmetry in GF peaks (p<0.01). Altogether, this analysis carried on the asymmetry of GF peaks, suggests that the scheme of a smooth superposition of automatic and voluntary processes does not hold at the highest movement frequencies.

### Experiment 2

**Figure 7:**
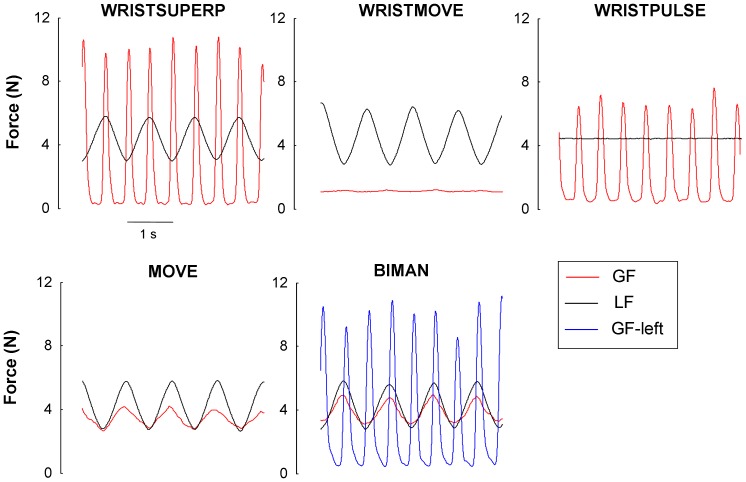
Typical trials in each experimental condition of Experiment 2. The top row presents the three conditions in which the elastic band was attached directly to the wrist. The bottom row presents the MOVE and the BIMANUAL condition in which the elastic band was connected to the hand-held object. All trials were performed by the same participant. Movement and squeezing frequency was set respectively at 1 Hz and 2 Hz.

**Task performance.** Typical trials by the same participant in each experimental condition are displayed in Figure 7. The analysis of LF signals in the WRIST-SUPERP, WRIST-MOVE, BIMANUAL and MOVE tasks showed that participants produced rather adequate movement amplitude. Averaged across frequencies and tasks, the mean group amplitude of LF was 3.11 N, and mean LF was 4.43 N. Within-cycle fluctuations in LF during the WRIST-PULSE task were below 0.1 N meaning that participants had the hand fixed. Concerning the timing aspect, the mean absolute deviation from the prescribed frequency intended for grip force or arm movement was 1.2±0.7% (error computed across all participants and all trials). Overall those values suggest that participants complied well with the instructions.

**Figure 8:**
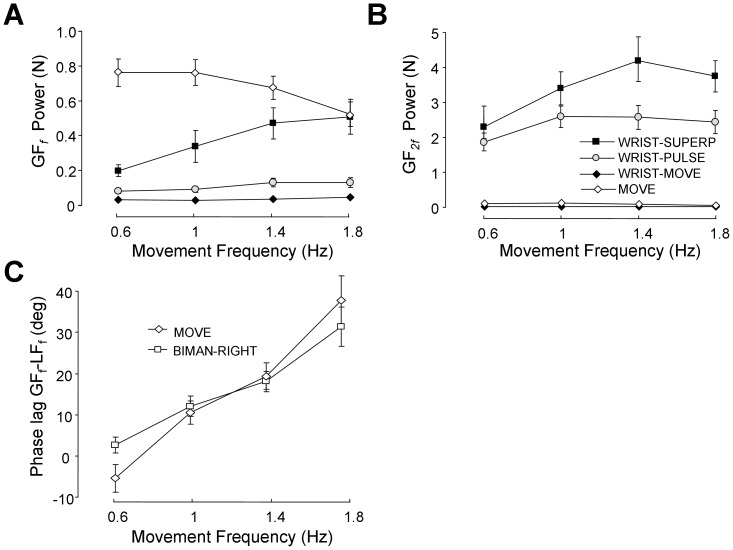
Spectral analysis of grip force signals in Experiment 2. A. Power of grip force at movement frequency (GF*_f_*) in all the WRIST tasks and the MOVE one. B. Same as A but for the power of grip force at twice the movement frequency (GF*_2f_*). C. Phase lag between grip force and load force at movement frequency during the MOVE and BIMAN tasks. A positive phase lag indicates that GF precedes LF. For all panels, data are averaged across participants, with error bars representing the standard error of the mean.

**Power spectral analyses of GF.** Figure 8A presents the mean group power of GF*_f_* as a function of *f* when the elastic was disconnected from the object and attached to the wrist. For comparison purposes, this analysis also included the MOVE task. Two-way ANOVA with *task* (4 levels) and *f* (4 levels) showed a main effect of *task* (3,57) = 36.57, p<0.001), but no effect of *f* (F(3,57) = 1.83, p = 0.15). As expected, the post-hoc indicated that GF*_f_* was greater in MOVE compared to all the other WRIST conditions. However, one can notice that during the WRIST-SUPERP task, GF*_f_* increased substantially as *f* increased. In contrast during the MOVE task, GF*_f_* decreased with *f* (as in Experiment 1). This led to a significant *f* by *task* interaction (F(9,171) = 7.57, p<0.001). The post-hoc analysis showed significant difference between the MOVE and WRIST-SUPERP tasks at all movement frequencies excepted at *f* = 1.8 Hz. Overall this spectral analysis showed that our procedure was successful in minimizing GF*_f_* during all WRIST conditions, excepted during WRIST-SUPERP at the highest frequency. Lastly, two-way ANOVA comparing the magnitude of GF*_f_* in MOVE during Experiment 1 and 2 (over the 4 movement frequencies in common) showed no main effects of *experiment* (F(1,38) = 0.01; p = 0.99) as well as no *f* by *experiment* interaction (F(3,114) = 0.57; p = 0.63), thereby suggesting that the two groups of participants had rather similar GF control.

Figure 8B presents the mean group power of GF_2*f*_ as a function of *f.* Two-way ANOVA with *task* and *f* showed a main effect of *task* (F(3,57) = 69.30, p<0.001) such that, as expected GF_2*f*_ was much greater in WRIST-SUPERP and WRIST-PULSE compared to WRIST-MOVE and MOVE. However there was also a main effect of *f* (F(3,57) = 13.44, p<0.001) and an interaction F(9,171) = 9.71, p<0.001). The post-hoc of the interaction indicated that, although GF_2*f*_ in the WRIST-PULSE and WRIST-SUPERP tasks was identical at 0.6 Hz, their magnitude diverged substantially as *f* reached 1.0 Hz (p<0.001). Overall, this analysis showed that despite our effort to minimize the contribution of automatic grip force control, the magnification of the voluntary GF pulses persisted under WRIST-SUPERP.

**Phase relationship between GF and LF during MOVE and BIMAN.** Figure 8C presents the mean group phase lag between GF*_f_* and LF*_f_* in the MOVE and BIMAN tasks. It was hypothesized that if voluntary pulses by the left hand do not interfere with automatic GF control in the right hand, this phase lag should be similar in both tasks. As in Experiment 1, the phase lag in MOVE was close from 0 at low movement frequency, and then increased with *f*. The same trend was observed in the BIMAN task. Indeed two-way ANOVA showed a main effect of *f* (F(3,57) = 50.10; p<0.001), but no effect of *task* (F(1,19) = 0.05; p>0.05) or interaction (F(3,57) = 2.10; p = 0.11). Overall this analysis suggests that voluntary pulses produced by the left hand did not interfere with automatic GF control in the right hand.

**Asymmetry of GF peaks during WRIST-SUPERP and BIMAN.** During WRIST-SUPERP, the magnitude of GF peaks in the vicinity of LF max tended to be larger than the one in the vicinity of LF min (GF_max1_  =  9.34 versus GF_max2_ = 9.07 N). However two-way ANOVA failed to reveal a main effect of *peak* (F(1,19) = 3.37, p = 0.08) or *peak* by *f* interaction (F(3,57) = 2.25, p = 0.09). As in Experiment 1, there was a main effect of *f* indicating that GF peaks magnitude increased with *f* (F(3,57) = 145.1, p<0.001). The analysis of GF peaks produced by the left hand during the BIMAN task led to rather similar results. Two-way ANOVA failed to reveal a main effect of *peak* (F(1,19) = 2.76, p = 0.07; GF_max1_  =  9.32 versus GF_max2_ = 9.59 N) or *peak* by *f* interaction (F(3,57) = 0.88, p = 0.45), but the main effect of *f* was significant (F(3,57) = 6.71, p<0.001). Altogether, these two control tasks provide no reliable evidence of GF peaks asymmetry, or switch in GF peaks asymmetry that could account for what was found in SUPERP during Experiment 1.

## Discussion

### Superposition of automatic and voluntary grip force control at low movement frequency

The goal of the current study was to investigate the extent to which automatic and voluntary grip force control can operate independently by means of a task that required to simultaneously oscillate an object and squeeze it at each movement reversal. The results brought by the first experiment showed that the scheme of a linear superposition of automatic and voluntary grip force control held at low movement frequency. Indeed, for slow movements, all the predictions formulated in the introduction (see Figure 1) were satisfied. These findings fit well with the seminal study of Flanagan and Wing [29] in which participants were required to oscillate a hand-held object more firmly. By investigating a novel paradigm in which voluntary GF control is more dynamical and challenging (i.e. participants being required to initiate grip force pulses at specific moments), the current study further documents the relative independence of voluntary and automatic GF control. Overall it is tempting to conclude that these two processes operate at different hierarchical levels. This possibility is in resonance with several neuroimaging and clinical studies showing that these two aspects of grip force control are mediated by different neural circuits. For instance using fMRI techniques it has been shown repeatedly that, in contrast to voluntary squeezing, automatic grip-load force coupling (as seen during object manipulation) involves specific brain regions such as the cerebellum and/or the posterior parietal cortex ([36]–[38]). Furthermore, although basal ganglia have been shown critical for the voluntary control of grip force (for a review see [39]), automatic aspects of grip force control are preserved in Parkinson patients [40]–[42]. Finally it is worth noting that the lack of interaction between automatic and voluntary grip force control during our bimanual task is also consistent with this scheme. This latter observation seems to contrast with previous studies showing some bimanual effects on GF-LF coupling [32], [43]–[45], but the fact that in all these studies both hands were concerned only with the automatic aspects of grip force control could be critical.

### Interactions between automatic and voluntary grip force control at high movement frequency

Despite converging evidences suggesting that voluntary and automatic grip force control operate at different hierarchical levels, the current study also showed the presence of interaction effects as movement frequency increased. A first observation brought by Experiment 1 was the magnification of GF oscillations. Concerning the magnification of voluntary ones, thanks to Experiment 2, we have shown that this effect persisted even when the elastic band was disconnected from the object and attached to the wrist. The fact that the magnification of the voluntary component was very similar in SUPERP and WRIST-SUPERP (respectively +48% and +43% over movement frequencies tested in common) suggests that superposition was not directly responsible for this effect. A first possibility could be a motoric effect known as *'spill over effect*' in which voluntary muscle activity tends to diffuse (involuntarily) to other neighbouring muscles [46]. One could envisage that higher recruitment in arm muscles with movement frequency was accompanied by higher recruitment in hand muscles, leading ultimately to higher GF pulses. A second possibility could be a perceptual effect. In order to synchronize adequately GF pulses with the beeps of the metronome, somatosensory feedback from the fingertips was presumably critical [47], [48]. However, as movement frequency increased, the flow of afferent information arising from the arm motion was likely to make finger actions less salient [49]. As a result, magnifying GF pulses may have been a solution to maintain the saliency of finger actions. Concerning the magnification of automatic GF oscillations, this effect is more intriguing. Indeed, despite our success in reducing the automatic component during WRIST-MOVE, this unnecessary component resumed during WRIST-SUPERP, up to a point where its magnitude was comparable to MOVE (see Figure 8A). At this stage the persistence and the magnification of this automatic component is still unclear, but the fact that we managed to keep this component silent during WRIST-PULSE and WRIST-MOVE suggests that oscillatory arm movements, in conjunction with voluntary GF pulses, are mandatory for this effect. It is concluded that, as for the voluntary component, superposition is not directly responsible for the magnification of automatic GF oscillations.

**Figure 9:**
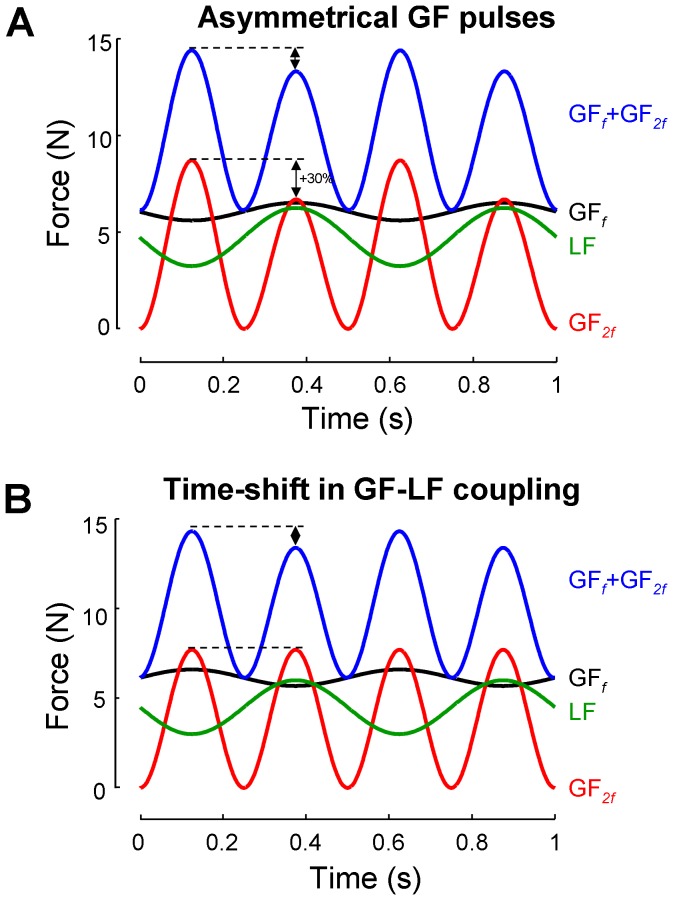
Two possible scenarios accounting for the shift in GF peaks asymmetry at high movement frequency. A. Scenario in which the voluntary component was altered. The asymmetry between successive voluntary GF pulses is set at 30%. B. Scenario in which the automatic component was altered. The automatic GF component is shifted with respect to the LF by 180°. In both examples, movement frequency was set at 2 Hz, and the power of each GF component was based on mean group data. See text for further details.

A second finding brought by Experiment 1 was the change in GF peaks asymmetry when movement frequency went beyond 1.6 Hz (see Figure 6C & 6D). In Figure 9, we proposed two extreme scenarios that could account for this switch in behaviour. In the first scenario (panel A) it is proposed that the switch in GF peaks asymmetry results from an alteration in the voluntary component (GF*_2f_*) such that voluntary pulses produced under high elastic load become smaller than the ones produced under low elastic load. Based on mean group data, this scheme suggests that a 30% asymmetry in voluntary GF peaks magnitude is necessary to account for a switch in GF peaks in overall GF. In the second scenario (panel B), it is suggested that the switch in GF peaks asymmetry results from a shift in the timing between the automatic component (GF*_f_*) and the load (180° in this example). On the one hand the results brought by the bimanual task do not speak in favour of an alteration of the voluntary component as there was no obvious asymmetry or switch in GF peaks asymmetry. On the other hand the second scenario is in resonance with the results of Experiment 1 showing that the temporal relationship between the automatic GF component and the load force was changed, but the alteration reported in Figure 5C is smaller than the one envisaged in Figure 9B (90° vs 180°). Overall at this stage, despite additional tests, it remains difficult to state which from the voluntary and automatic components is most responsible for the change in GF peaks asymmetry and intermediate scenarios should certainly not be excluded.

### Limitations of the study

Our combined task in which subjects had to simultaneously oscillate the object and squeeze it at each movement reversal was a nontrivial task because it required some fine coordination between hand and arm actions plus it does not mimic an every-day activity. Although subjects were able to perform this combined task at various movement frequencies, their ability to complete the task in a single attempt decreased with movement frequency. All in all, it is possible that, in addition to the other mechanisms envisaged previously, the increase in difficulty with movement frequency may have contributed to the emergence of interaction effects between automatic and voluntary GF control. Ultimately, one may question whether similar frequency dependent effects would have been obtained if subjects had received more extensive practice.

### Concluding comments

Overall the current study showed that voluntary and automatic GF control function independently when arm movement is performed at low frequencies. This finding is interpreted as an evidence that these two processes operate at different hierarchical levels. Concerning the presence of interaction effects at higher movement frequencies, some of these effects (GF magnification) may not directly stem from the superposition of voluntary and automatic GF control, however their precise origin remain to be found and future studies will have to investigate whether these effects persist with more extended practice. At a more general level, this study further documents the superposition principle in human grasp control [50], , a concept suggesting that grasping can be divided into multiple, and independently controlled components that summate to form a complete command [52]. Finally, despite restrictions with respect to temporal constraints, this study extends the ability of the nervous system to flexibly combine automatic and voluntary control beyond basic functions such as breathing, swallowing, and walking [1], [2], [5], [7], [8].
